# Vascular Involvement in Behçet’s Disease: A Retrospective Analysis of Qatar Behçet’s Cohort

**DOI:** 10.2147/OARRR.S567674

**Published:** 2026-05-04

**Authors:** Rawan Saleh, Samar AlEmadi, Nabeel Abdulla, Israa AlSheikh, Mousa Ahmad Alhiyari, Thurayya Arayssi

**Affiliations:** 1 Rheumatology Department, Hamad Medical Corporation, Doha, Qatar

**Keywords:** Behçet’s syndrome, vasculitis, thrombosis, middle east, immunosuppression

## Abstract

**Background:**

Behçet’s syndrome (BS) is a chronic systemic inflammatory disorder with vascular involvement representing a severe complication. Despite its high prevalence along the Silk Route, data on vascular Behçet’s in Middle Eastern populations remain limited.

**Purpose:**

This study evaluates the prevalence, clinical features, and treatment outcomes of vascular BS in Qatar’s multinational cohort.

**Patients and Methods:**

A retrospective analysis was conducted on 82 BS patients (2016–2024) meeting the International Criteria for Behçet’s Disease (ICBD). Vascular involvement was confirmed via imaging (Doppler, CTA/MRA). Demographics, clinical manifestations, and treatment responses were characterized using descriptive analyses, and intervariable associations were statistically examined.

**Results:**

Vascular involvement was identified in 20.7% (17/82) of patients, with a male predominance (76.5%) and earlier diagnosis among Arab patients (29.3 vs. 44.3 years, p=0.02). Multivascular involvement was the most common pattern (35.3%), followed by isolated venous thrombosis (29.4%) and isolated arterial involvement (11.8%). Overall, venous disease was the predominant vascular manifestation, occurring either as isolated venous thrombosis or as part of combined arterial–venous involvement. The favorable clinical outcomes observed with corticosteroid–immunosuppressant combination therapy strongly support the early initiation of aggressive immunomodulatory treatment in patients with vascular Behçet’s disease.

**Conclusion:**

In Qatar, about 1 in 5 patients with Behçet’s disease show signs of vascular involvement, with notable differences based on ethnicity and gender. Venous and multivascular involvement are more common, making comprehensive imaging essential. Immunosuppression, particularly steroid-biologic combinations, appears superior to anticoagulation alone. These findings highlight the need for region-specific management protocols in this high-risk population.

## Introduction

Behçet’s syndrome (BS) is a chronic, relapsing, multisystem inflammatory disorder defined by recurrent oral and genital ulcers and variably associated ocular, musculoskeletal, vascular, gastrointestinal, and neurologic involvement. Its pathogenesis is multifactorial, involving genetic susceptibility—most notably HLA-B51—environmental triggers, and dysregulated innate immune mechanisms.^1–3^

BS exhibits a distinct geographic distribution, with the highest prevalence along the historical Silk Route, particularly in Turkey, Iran, and Japan, and lower yet clinically relevant rates throughout the Middle East and Mediterranean regions.^4^,^5^

Although not the most common manifestation, vascular involvement constitutes one of the most serious complications of BS. Behçet-related vasculitis is characterized by neutrophilic inflammation, endothelial dysfunction, and a prothrombotic milieu, permitting both arterial and venous involvement across all vessel sizes.^3^,^6^ The prevalence of vascular disease varies widely, ranging from 10% to 30% across populations.^3^,^7–9^ Vascular manifestations may precede typical mucocutaneous symptoms in up to one-third of cases, creating diagnostic uncertainty and necessitating heightened suspicion, particularly in young patients from endemic regions.^3^,^10^,^11^ Vascular BS is further associated with increased morbidity due to thrombosis, aneurysm formation, and disease relapse, and with higher mortality compared with non-vascular forms.^6^,^12^ Cohort data suggest that while overall mortality in BS is only modestly elevated relative to the general population, it rises substantially in young men and in patients with major vascular complications.^13^,^14^ Management of vascular BS primarily relies on immunosuppressive therapy, with anticoagulation used cautiously due to limited evidence and bleeding risk. Arterial involvement—particularly aneurysms—typically requires high-dose corticosteroids with additional immunosuppressants. TNF-α inhibitors are recommended for severe or refractory vascular disease, including pulmonary arterial involvement. Surgical or endovascular interventions are reserved for life-threatening complications given the elevated risk posed by active inflammation.^15^

Qatar has a unique multi-national population, which makes it easier to study the problem across different ethnic groups. This study aims to determine the prevalence, clinical spectrum, and outcomes of treatment of vascular Behçet’s syndrome in the population of Qatar, focusing on ethnicity differences in presentation and response to treatment.

## Materials and Methods

### Study Population and Design

We conducted a retrospective cohort study at Hamad Medical Corporation (HMC), Qatar’s main tertiary care center, which provides services to the majority of the country’s population. The study included all patients aged 18 and above who had a confirmed diagnosis of Behçet’s syndrome based on the International Criteria for Behçet’s Disease (ICBD) criteria and were regularly followed in the rheumatology clinics between July 1, 2016, and April 1, 2024. Patients were excluded if they had incomplete medical records or if their diagnosis did not meet the ICBD classification criteria. In our cohort, 82 patients met the diagnostic criteria for Behçet’s syndrome, of whom 17 (20.7%) exhibited vascular involvement.

### Data Collection and Variables

Patient data were obtained from the Cerner electronic health record system, which has been fully integrated across all HMC facilities since July 2016. We used a standardized data collection form to systematically record demographic information (including age, gender, and ethnicity), key clinical features (such as oral and genital ulcers, skin lesions, ocular and neurological involvement), and details of any vascular manifestations.

The diagnosis of vascular involvement was confirmed through a combination of clinical assessment and imaging studies. Venous involvement was defined by the presence of objectively confirmed deep vein thrombosis (DVT), superficial venous thrombosis (SVT), or cerebral venous sinus thrombosis (CVST) using Doppler ultrasonography, computed tomography venography, or magnetic resonance venography. Arterial involvement included pulmonary artery aneurysms, aortic aneurysms, peripheral artery thrombosis, or other arterial pathologies documented by computed tomography angiography (CTA), magnetic resonance angiography (MRA), or conventional angiography. Cardiac involvement was defined by the presence of intracardiac thrombi or coronary artery disease confirmed by echocardiography or coronary angiography.

### Treatment and Outcome Assessment

The main outcome examined was the occurrence of vascular relapses, defined as any new or recurrent vascular event following the initial diagnosis. Relapses were identified through a combination of clinical assessment during routine follow-up visits and imaging studies, performed when new symptoms or clinical suspicion warranted further evaluation.

Our Patients had regular rheumatology clinic follow-ups, typically every 3 to 6 months, or more frequently in cases of active disease. Clinical notes, Inflammatory markers, and imaging reports were reviewed to monitor disease activity, detect relapses, and assess treatment response.

### Statistical Analysis

We used descriptive statistics to summarize the demographic and clinical characteristics of the study population. Continuous variables were presented as mean ± standard deviation (SD) or as median with interquartile range (IQR), depending on whether the data followed a normal distribution. Categorical variables were reported as frequencies and percentages.

To compare patients with and without vascular involvement, we used Pearson’s chi-square test or Fisher’s exact test for categorical variables, as appropriate. For continuous variables, either the Student’s *t*-test or the Mann–Whitney *U*-test was applied, depending on the distribution of the data.

To explore factors associated with vascular involvement and relapse, we first evaluated each variable individually using the appropriate statistical tests. Variables that demonstrated a p-value <0.1 in these initial analyses were considered for further multivariable assessment. All statistical analyses were carried out using IBM SPSS Statistics, version 26 (Armonk, NY, USA), with a two-tailed p-value <0.05 considered statistically significant.

### Ethical Considerations

The study protocol was reviewed and approved by the Institutional Review Board (IRB) of Hamad Medical Corporation. Given the retrospective nature of the study, the requirement for informed consent was waived by the IRB. Patient data were anonymized and de-identified prior to analysis to ensure confidentiality.

## Results

### Demographic Characteristics (Table 1)

We observed vascular involvement in 17 out of 82 patients with BS, corresponding to a prevalence of 20.7%. The majority of these cases were male (82.3%). Most patients (76.4%) were of Arab descent. The average age at diagnosis in our study was 31.9 years. Interestingly, Arab patients were diagnosed significantly earlier, with a mean age of 29.3 years, compared to 44.3 years among non-Arab patients (p = 0.02). This ethnic difference in age at diagnosis may be influenced by the geographic and demographic characteristics of the study population, where Arab individuals represent the majority.

**Table 1:** Demographic Characteristics (n=17) Including Age, Gender, Ethnicity, and Age at Diagnosis

Characteristic	Category	n (%) or Mean ± SD	Range
Gender	Male	14 (82.3%)	–
	Female	3 (17.6%)	–
Ethnicity	Arab	13 (76.4%)	–
	Asian	3 (17.6%)	–
	African	1 (5.9%)	–
Age (years)	Current	41.2 ± 12.9	18–64
	Diagnosis	31.9 ± 11.8	17–59

Although the difference was not statistically significant, female patients in our cohort tended to be diagnosed later than male patients (mean age: 37.8 vs. 30.2 years, p = 0.21). While this trend may simply reflect sampling variation, it could also suggest underlying gender-related diagnostic delays or variations in disease expression, an area that would benefit from further investigation in larger, more diverse studies.

### Vascular Involvement Pattern (Table 2)

Among the 17 patients with vascular involvement, isolated venous disease was observed in 29.4% (5/17), with deep vein thrombosis (DVT) accounting for 80% of these cases. Isolated arterial (11.8%) and isolated cardiac (5.9%) involvement were less common. Multivascular involvement was seen in 35.3% (6/17) of patients, most frequently as combined arterial–venous disease (17.6%). These findings indicate that although isolated venous thrombosis is the most common single-category presentation, venous involvement overall is the predominant vascular manifestation in Behçet’s syndrome, given its frequent occurrence in multivascular patterns.

**Table 2:** Vascular Involvement Patterns (n=17)

Category	Cases (n)	Prevalence (%)	95% CI
Arterial alone	2	11.8%	2.1–34.9%
Venous alone	5	29.4%	12.4–54.7%
Cardiac alone	1	5.9%	0.3–27.3%
Arterial + Venous	3	17.6%	4.7–43.6%
Arterial + Cardiac	1	5.9%	0.3–27.3%
Venous + Cardiac	1	5.9%	0.3–27.3%
All three types	2	11.8%	2.1–34.9%

### Comparison of Demographic and Clinical Characteristics Between Patients with and without Vascular Involvement (Table 3)

A comparative analysis of demographic and clinical characteristics between BS patients with and without vascular involvement revealed no statistically significant differences in age or major clinical features (p > 0.05 for all comparisons).

**Table 3:** Statistical Comparison: Vascular vs. Non-Vascular Involvement

Feature	Vascular (n=17)	Non-Vascular (n=65)	p-value	Significance
Genital lesions	10 (58.8%)	38 (58.5%)	0.98	NS
Oral aphthosis	17 (100%)	62 (95.4%)	0.36	NS
Neurological manifestations	2 (11.8%)	6 (9.2%)	0.74	NS
Ocular lesions	7 (41.2%)	33 (50.8%)	0.47	NS
Positive pathergy test	2 (11.8%)	5 (7.7%)	0.59	NS
Skin manifestations	10 (58.8%)	34 (52.3%)	0.63	NS
Smoking status	4 (23.5%)	11 (16.9%)	0.52	NS
Age at diagnosis (years)	31.8 ± 12.9	34.5 ± 9.7	0.39	NS

Despite the absence of significant differences, several clinically relevant trends were observed. Patients with vascular involvement exhibited slightly higher rates of neurological involvement (11.8% vs. 9.2%) and smoking (23.5% vs. 16.9%). Conversely, patients without vascular involvement had a higher incidence of ocular lesions (50.8% vs. 41.2%).

### Treatment Outcomes

The majority of patients received a combination treatment regimen including corticosteroids (such as prednisone or its alternatives), immunosuppressive medications (including azathioprine, methotrexate, cyclophosphamide, or TNF inhibtors), and anticoagulants (primarily warfarin or low-molecular-weight heparin). The treatment decisions were guided by disease severity and the type and extent of vascular involvement as determined by the treating rheumatologist.

Among patients with vascular involvement, treatment with immunosuppressants, in combination with corticosteroids, was significantly associated with a reduced risk of relapse (*p* = 0.046). Anticoagulation therapy showed no apparent effect on relapse rates. Larger, controlled studies are necessary to validate these results and to clarify the individual contributions of steroids and immunosuppressants.

## Discussion

Our study provides the first comprehensive evaluation of vascular Behçet’s syndrome (BS) in Qatar, offering new insights with important clinical implications. The observed prevalence of vascular involvement, 20.7%, underscores the significance of this complication in our population and is comparable to rates reported along the Silk Route. For instance, the UAE cohort reported vascular involvement in approximately 20–30% of patients, with males showing higher rates of major organ involvement,^16^ while the Omani study found vascular complications in 23% of patients, predominantly affecting younger males with shorter disease duration,^17^ These findings collectively emphasize the need for clinical vigilance regarding vascular complications in Behçet’s patients across the region.

A pronounced male predominance was evident in our vascular cohort (76.5%), aligning with regional patterns reported in KSA,^18^ where males comprised 70% of the study population and demonstrated higher rates of vascular and neurological manifestations. Similarly, both the UAE and Oman studies documented a higher prevalence of vascular complications among men. This gender disparity may result from androgen-mediated immune modulation, which exacerbates neutrophilic hyperreactivity characteristic of Behçet’s vasculitis, diagnostic bias with men presenting more severe symptoms, and potential endothelial protective effects of estrogen in women. Collectively, these observations suggest that male sex is a consistent risk factor for vascular involvement in Behçet’s disease in Middle Eastern populations.

Differences in age at diagnosis between ethnic groups in our cohort were notable and clinically relevant. Arab patients were diagnosed significantly earlier than non-Arabs (29.3 vs. 44.3 years), likely reflecting Qatar’s demographic composition, with many Arab patients having lifelong healthcare access and heightened disease awareness. Non-Arab patients, often expatriates, may experience delayed diagnosis despite disease onset at a similar age. These findings mirror the Omani study, where younger patients were more likely to develop vascular events, emphasizing that early recognition in younger adults—particularly males—is critical for timely intervention. Such demographic distinctions highlight the need for stratified monitoring strategies based on ethnicity and age.

The pattern of vascular involvement observed in our cohort provides additional insights into disease pathophysiology. Isolated venous thrombosis, predominantly deep vein thrombosis (DVT), was the most common manifestation (29.4%), with multivascular involvement present in 35.3% of patients. This aligns with previous reports from the UAE and KSA, which also documented venous predominance and frequent involvement of multiple vascular territories. In Oman, a similar trend was noted, with mixed arterial–venous involvement in 44% of vascular patients and pulmonary artery aneurysms observed in a subset. These findings suggest that Behçet’s vasculitis preferentially damages venous endothelium while potentially involving multiple vascular beds, supporting the need for comprehensive imaging in high-risk patients.

Our study has limitations, including its retrospective design, which may have led to underreporting of milder vascular events, and a relatively small sample size that limits statistical power for subgroup analyses. Additionally, as a single-center study, our findings may not fully represent the broader diversity of Behçet’s patients in the Gulf region. Nevertheless, the integration of data from a multinational referral population, combined with regional comparisons, provides a robust framework to contextualize our results and offers valuable guidance for clinical practice in Qatar and similar Middle Eastern populations.

## Conclusion

This study provides the first comprehensive analysis of vascular involvement in Behçet’s Disease in Qatar’s diverse population, offering important insights for clinical practice. Vascular involvement was observed in 20.7% of patients, confirming it as a significant complication in Middle Eastern Behçet’s populations, consistent with other regional and Silk Route cohorts.

Male sex, Arab ethnicity, and younger age at diagnosis emerged as important risk factors. These findings highlight the need for heightened vigilance in young male patients presenting with unexplained thrombosis or aneurysms.

Finally, the pattern of vascular disease, characterized by frequent venous thrombosis and multivascular involvement, underscores the importance of comprehensive vascular assessment. Treatment outcomes emphasize the central role of immunosuppressive therapy, with evidence supporting early and aggressive immunomodulation over conventional anticoagulation alone.

Overall, these findings enhance understanding of vascular Behçet’s in the Middle East and support tailored diagnostic and therapeutic strategies.

## Abbreviations

BS, Behçet’s Syndrome; DVT, Deep Vein Thrombosis; SVT, Superficial Venous Thrombosis; CVST, Cerebral Venous Sinus Thrombosis; CTA, Computed Tomography Angiography; MRA, Magnetic Resonance Angiography; ICBD, International Criteria for Behçet’s Disease; HMC, Hamad Medical Corporation; IRB, Institutional Review Board; TNF, Tumor Necrosis Factor; SPSS, Statistical Package for the Social Sciences; SD, Standard Deviation; IQR, Interquartile Range; NY, New York; IBM, International Business Machines.
